# A prospective study of cancer risk among Agricultural Health Study farm spouses associated with personal use of organochlorine insecticides

**DOI:** 10.1186/s12940-017-0298-1

**Published:** 2017-09-06

**Authors:** Lydia M. Louis, Catherine C. Lerro, Melissa C. Friesen, Gabriella Andreotti, Stella Koutros, Dale P. Sandler, Aaron Blair, Mark G. Robson, Laura E. Beane Freeman

**Affiliations:** 1 Occupational and Environmental Epidemiology Branch, Division of Cancer Epidemiology and Genetics, National Cancer Institute, National Institutes of Health, Bethesda, MD USA; 2 School of Public Health, Rutgers University, Piscataway, NJ USA; 3 Epidemiology Branch, National Institute of Environmental Health Sciences, National Institutes of Health, Research Triangle Park, Durham, NC USA; 4 School of Environmental and Biological Sciences, Rutgers University, New Brunswick, NJ USA

**Keywords:** Agricultural Health Study, Cancer, Farm spouses, Organochlorine, Pesticide

## Abstract

**Background:**

Organochlorine insecticides (OCs) have historically been used worldwide to control insects, although most have now been banned in developed countries. Evidence for an association between OC exposures and cancer predominantly comes from occupational and population based-studies among men. We evaluated the association between the use of specific OCs and cancer among the female spouses of pesticide applicators in the Agricultural Health Study.

**Methods:**

At enrollment (1993–1997), spouses of private applicators in the cohort provided information about their own use of pesticides, including seven OCs (aldrin, chlordane, dieldrin, DDT, heptachlor, lindane, and toxaphene), and information on potential confounders. We used Poisson regression to estimate relative risks (RRs) and 95% confidence intervals (CIs) for cancers (*n* ≥ 3 exposed cases) reported to state cancer registries from enrollment through 2012 (North Carolina) and 2013 (Iowa), and use of the individual OCs, as well as use of any of the specific OCs.

**Results:**

Among 28,909 female spouses, 2191 (7.58%) reported ever use of at least one OC, of whom 287 were diagnosed with cancer. Most cancers were not associated with OC use. Risk of glioma was increased among users of at least one OC (N_exposed_ = 11, RR = 3.52, 95% CI 1.72–7.21) and specifically among lindane users (N_exposed_ = 3, RR = 4.45, 95% CI 1.36–14.55). Multiple myeloma was associated with chlordane (N_exposed_ = 6, RR = 2.71, 95% CI 1.12–6.55). Based on 3 exposed cases each, there were also positive associations between pancreatic cancer and lindane, and ER-PR- breast cancer and dieldrin. No other associations with breast cancer were found.

**Conclusions:**

Overall, there were some associations with OC use and cancer incidence, however we were limited by the small number of exposed cancer cases. Future research should attempt to expand on these findings by assessing environmental sources of OC exposures, to fully evaluate the role of OC exposures on cancer risk in women.

## Background

Organochlorine insecticides (OCs) are a class of chlorinated hydrocarbon insecticides historically used worldwide in agriculture. Some are still used in some developing countries for the control of vector borne illnesses [1–3]. OCs were first introduced in the United States in the 1940s and were used widely in agriculture and pest control through the 1960s. Due to their environmental persistence and ability to bioacccumulate, most OCs were banned for use in the U.S. during the 1970s and 1980s. Lindane and endosulfan were banned more recently in 2006 and 2010, respectively [4–6]. The International Agency for Research on Cancer (IARC) has reviewed the carcinogenicity of fifteen OCs. Of these, lindane [7] and pentachlorophenol (PCP) [8] were classified as Group 1, carcinogenic to humans; dichlorodiphenyltrichloroethane (DDT) [7], aldrin [8] and dieldrin [8] as Group 2A, probably carcinogenic to humans; 2,4,6-trichlorophenol (TCP) [8], chlordane [9], chlordecone [10], heptachlor [9], hexachlorocylcohexanes (HCH) [10], hexachlorobenzene (HCB) [9], mirex [10], and toxaphene [9] as Group 2B, possibly carcinogenic to humans; and endrin [10] and methoxychlor [10] classified as Group 3, not classifiable as to their carcinogenicity to humans.

The strongest epidemiologic evidence for a link between OC exposures and cancer risk comes from occupational and population-based studies of non-Hodgkin lymphoma (NHL) [11–19]. Previous analyses of licensed pesticide applicators in the Agricultural Health Study (AHS) found significant positive exposure-response trends for lindane and DDT use with NHL [12], and chlordane and heptachlor use with leukemia [20]. In a pooled analysis of Canadian and U.S. based case–control studies, self-reported ever use of DDT was significantly associated with multiple myeloma (MM) [17]. The strongest evidence for associations with cancer at other sites comes from case–control studies of liver and testicular cancers [21–24]. DDT, dichlorodiphenyldichloroethylene (ρ, ρ’ -DDE), and β-HCH have been associated with hepatocellular carcinoma [21–23], and testicular germ cell tumors have been significantly associated with prediagnostic serum DDE and chlordane metabolites [24]. There is also evidence for significant positive associations with OC exposures and cancers of the prostate [25], skin (cutaneous melanoma) [20], lung [20], rectum [20], and pancreas [26, 27]. An evaluation of pesticides and glioma reported no association with OCs [28].

In addition, potential associations between OCs and hormonally-mediated cancers, particularly female breast cancer, are of concern due to the endocrine disrupting properties of OCs [29–38]. While two studies [34, 35] have suggested an association with dieldrin, most studies are null [29, 32, 33, 36–39]. However, several reports provide evidence for an increased risk of breast cancer in adulthood with early life exposure to OCs [40–43]. The one study of endometrial cancer found no associations with OCs overall [44]. No studies to our knowledge have investigated associations with ovarian cancer. Although in vitro and in vivo studies suggest OCs may act as estrogen agonists or antagonists [45–48], the relationship between OCs and hormonally-mediated cancers among women remains unclear. In this paper, we examine the associations between multiple site-specific cancers and self-reported personal use of OCs among the female spouses of AHS applicators.

## Methods

### Study population and follow-up

The AHS is a prospective cohort that includes licensed private pesticide applicators (mostly farmers), and the spouses of private pesticide applicators residing in Iowa and North Carolina. The AHS has been previously described in detail [49]. Pesticide applicators were recruited from 1993 to 1997 when obtaining a license to apply restricted-use pesticides. Private pesticide applicators who reported being married at the time of enrollment were given questionnaires to be completed by their spouses. The spouses (*n* = 32,345) of these private pesticide applicators are the focus of this study. The Spouse Enrollment questionnaire elicited information on demographic and lifestyle factors, family and personal medical histories, farm exposures, and agricultural activities, including the application or mixing of specific pesticides. In addition, 60.0% of the spouses in this analysis also completed the Female and Family Health questionnaire which focused on reproductive health histories. The study protocol was approved by all relevant institutional review boards. Study questionnaires are publicly available (https://aghealth.nih.gov/collaboration/questionnaires.html).

Cancer incidence was assessed regularly via linkage with the North Carolina and Iowa state cancer registries. Mortality incidence was assessed through regular linkage with state mortality registries and The National Death Index. Cancer sites were classified according to the *International Classification of Diseases for Oncology, 3rd revision* (World Health Organization). For NHL, we followed the Surveillance Epidemiology and End Results (SEER) lymphoma coding scheme [50].

### Exposure assessment and questionnaires

Spouses of private pesticide applicators were enrolled in the AHS from 1993 to 1997, during which time they reported their lifetime never/ever personal use of fifty pesticides including past use of seven OCs (aldrin, chlordane, dieldrin, DDT, heptachlor, lindane and toxaphene). For each pesticide, they were asked ‘During your lifetime, have you ever personally mixed or applied [pesticide]? (Includes pesticides used for farm use, commercial application and personal use in your home or garden)’. Participants who indicated ever use of at least one of these seven OCs were classified as having personally used ‘any OC’, whereas those who indicated never use of any of these OCs were classified as never having personally used ‘any OC’. Otherwise, participants were considered to be missing as to their ‘any OC’ use. In the following analysis, the term ‘any OC’ will be used to refer to the ever personal use of at least one of these seven OCs.

### Statistical analysis

For this analysis we excluded, the 219 male spouses, women who were diagnosed with cancer prior to study enrollment (*n* = 905), those with zero or missing person years of follow-up (*n* = 161), and those missing information on use for all seven OCs (*n* = 2146), leaving 28,909 female spouses in our analytic cohort. Relative risks (RR) and 95% confidence intervals (95% CI) were calculated for risk of cancer among ever users, compared to never users, using multivariable *Poisson* regression in SAS version 9.3 (SAS Institute, Inc., Cary, N.C.). We evaluated all cancer sites with at least three exposed cases for associations with each of the seven individual OCs and for the use of any OC as defined previously. Person-time accrued from the date of study enrollment until date of death, cancer diagnosis, movement out of state or last study-follow-up (December 31, 2012 and December 31, 2013 for North Carolina and Iowa respectively), whichever was earliest. For the evaluation of uterine and ovarian cancers, person-time was censored at the time of hysterectomy or oophorectomy, where applicable.

All models were adjusted for age at enrollment (≤44 years, 45–54 years, 55–64 years, ≥ 65 years), educational attainment (high school degree or less, some college or college graduate, one or more years of graduate school), alcohol use (never, less than 1 drink per month, ≥ 1–3 per month), cigarette pack-years smoked as reported at enrollment (pack-year quartiles: Never, ≤ 6.75, 6.751–16.75, ≥ 16.751), and state of residence (Iowa or North Carolina). We considered the following additional confounders: BMI, race, family history of cancer, and ever use of any pesticide, but did not include them in our final models as they did not appreciably alter our results by ≥ 10%. For all cancer sites, mutually adjusting for individual non-OC and OC pesticides that were correlated at ρ ≥ 0.4 (i.e. aldrin and dieldrin (ρ = 0.43), aldrin and heptachlor (ρ = 0.42)) did not appreciably change our results and these adjustments were not included in our final models. Moreover, because dieldrin is also a biological metabolite of aldrin, we performed sensitivity analyses where ‘dieldrin metabolite’ (i.e. those farm spouses who personally used either aldrin or dieldrin) was modeled as the exposure. However, these analyses did not significantly alter our existing aldrin and dieldrin results, and were not included in our final analyses.

Additionally, we examined potential confounders known to be associated with specific cancer sites, such as total meat consumption (colon, rectum, stomach), sun sensitivity (melanoma), asbestos exposure (lung), autoimmune disorders, exposure to livestock and poultry, and benzene exposure (lymphohematopoeitic cancers); adjusting for these specific cancer-related covariates did not alter our results and were not included in our final models. All OC-exposed brain cancer cases were glioma subtypes; thus, we report associations here for glioma only. Breast cancers were examined overall, as well as by estrogen receptor (ER) and progesterone receptor (PR) status, where available. Female health and reproductive covariates at enrollment were also examined, with respect to breast, ovarian and uterine cancer, and included the following: menopausal status, ever use of oral contraceptives, ever use of estrogen-based hormone replacement therapy, ever use of progestin based hormone replacement therapy, age at menarche, and parity. These female reproductive covariates did not appear to significantly alter our results and thus were not included in our final models. Due to a lack of questionnaire information availability, we were unable to assess age at first live birth as a potential covariate. We conducted stratified analyses by several female health covariates, including menopausal status at enrollment (yes/no), ever use of oral contraceptives at enrollment (yes/no), ever use of hormone replacement therapy at enrollment (yes/no) and age at first menarche (≤ 12 years or below, > 12 years). Due to the small number of nulliparous women (*n* = 294), we were unable to evaluate risks among nulliparous women.

## Results

From enrollment through 2012/2013, the 28,909 female spouses contributed a total of 502,895 person-years of follow-up (Mean = 16.19 standard deviation +/− 3.8). Overall, 15,112 (52.3%) reported ever using any pesticide (data not shown), and 2191 (7.6%) reported ever use of any of the seven OCs included in the enrollment questionnaire. The most commonly reported OCs were chlordane (4.1%), DDT (3.55%) and lindane (1.5%), with less than 1% of participants reporting ever use of aldrin, heptachlor, toxaphene and dieldrin (Table 1). Among women who reported using any OC, 718 (32.8%) reported use of more than one OC. Ever OC users tended to be older, have higher BMIs, be from Iowa, and have a higher educational level than OC non-users. They were also more likely to have reported a family history of cancer, grown up on a farm, used oral contraceptives, and have an earlier onset of menarche. Additionally, 77.5% of OC users completed the Female and Family Health Questionnaire versus 60.5% of never OC users.

**Table 1:** Select characteristics of AHS farm spouses at enrollment with OC insecticide personal use information (*n* = 28,909)^a^

	Any OC use^b^				
	Never		Ever		
	*N* = 26,718	%	*N* = 2,191	%	*p* value^±^
Age at enrollment					
≤ 44	13,153	49.23	447	20.40	< .0001
45–54	6,677	24.99	771	35.19	
55–64	4,823	18.05	731	33.36	
≥ 65	2,065	7.73	242	11.05	
Race					
White	26,164	97.93	2,178	99.4	< .0001
Other	510	1.91	8	0.37	
Missing	44	0.16	5	0.23	
State of Residence					
North Carolina	8,739	32.71	632	28.85	0.0002
Iowa	17,979	67.29	1,559	71.15	
Educational Attainment					
High School or less	13,905	52.04	1,102	50.3	0.0015
Some College or College Graduate	8,662	32.42	679	30.99	
1 or more years of Graduate School	3,833	14.35	378	17.25	
Missing	318	1.19	32	1.46	
Body Mass Index					
0–24.99	11,756	40.67	902	41.17	< .0001
25.00–29.99	7,448	25.76	717	32.72	
≥ 30.00	4,298	14.87	440	20.08	
Missing	3,216	11.12	132	6.02	
Alcohol					
Never	12,042	45.07	939	42.86	0.2371
Less than once/month	7,099	26.57	603	27.52	
≥ 1–3 times per month	7,277	27.24	621	28.34	
Missing	300	1.12	28	1.28	
Cigarette Smoking (Pack-years)					
Never Smoker	18,820	70.44	1,493	68.14	0.0003
≤ 6.75	3,544	13.26	294	13.42	
6.751–16.75	1,752	6.56	127	5.80	
≥ 16.751	1,700	6.36	186	8.49	
Missing	902	3.38	91	4.15	
Family History of Cancer					
No/Missing	13,949	52.21	949	43.31	< .0001
Yes	12,769	47.79	1,242	56.69	
Grew up on a farm					
No	10,913	40.85	534	24.37	< .0001
Yes	15,579	58.30	1,643	74.99	
Missing^c^	226	0.85	14	0.64	
Menopause at Enrollment					
No	8,922	33.39	534	24.37	< .0001
Yes	6,777	25.36	1,127	51.44	
Unsure	245	0.92	22	1.00	
Missing^c^	10,774	40.32	508	23.19	
Number of Live Births					
0	235	0.88	29	1.32	< .0001
1 or 2	7,216	27.01	688	31.40	
> 2	7,526	28.17	887	40.48	
Missing^c^	11,741	43.94	587	26.79	
Oral Contraceptive Use					
Never	4,236	15.85	576	26.29	< .0001
Ever	11,722	43.87	1,106	50.48	
Missing^c^	10,760	40.27	509	23.23	
Age of first menarche					
12 years or less	7,099	26.57	788	35.97	< .0001
13 years	4,851	18.16	493	22.5	
14 years or greater	3,855	14.43	398	18.17	
Missing^c^	10,913	40.85	512	23.37	
OC Insecticides					
Overall OC (Any OC)	-	-	2,191	7.58	-
Chlordane	-	-	1,196	4.13	-
DDT	-	-	1,028	3.55	-
Lindane	-	-	430	1.47	-
Aldrin	-	-	235	0.81	-
Hepatchlor	-	-	222	0.77	-
Toxaphene	-	-	203	0.70	-
Dieldrin	-	-	105	0.36	-

*AHS* Agricultural Health Study, *RR* relative risks, *OC* organochlorines

^±^Chi Square test for homogeneity

^a^Excluded: *n* = 2146 with missing information for personal use of all OCs; *n* = 905 female spouses diagnosed with cancer prior to enrollment; *n* = 219 male spouses; *n* = 161 with missing or 0 person-years of follow-up leaving a total *n* = 28,909 female farm spouses

^b^Ever use of any of the seven OC insecticides

^c^From Female & Family Health Questionnaire responses

Any OC use was significantly associated with an increased risk of glioma (N_exposed_ = 11; RR = 3.52, 95% CI 1.72 to 7.21) (Table 2). Lindane use was significantly associated with an increased risk of glioma (N_exposed_ = 3, RR = 4.45 95% CI 1.36 to 14.55) and pancreatic cancer (N_exposed_ = 3, RR = 3.70 95% CI 1.15 to 12.0). Use of any OC was also associated with non-significantly elevated risks of stomach cancer (N_exposed_ = 5, RR = 2.61, 95% CI 0.96 to 7.11), and colon cancer (N_exposed_ = 28, RR = 1.19, 95% CI 0.80 to 1.75).

**Table 2:** RR and CIs^b^ for ever versus never use of OC insecticides, for all cancer sites^±^

	N _total_	Any OC^a^			Chlordane			DDT			Lindane			Aldrin			Heptachlor			Toxaphene			Dieldrin		
		N _exposed_	RR	95% CI	N _exposed_	RR	95% CI	N _exposed_	RR	95% CI	N _exposed_	RR	95% CI	N _exposed_	RR	95% CI	N _exposed_	RR	95% CI	N _exposed_	RR	95% CI	N _exposed_	RR	95% CI
All Cancer Sites^c^	3,204	287	0.96	0.85–1.08	160	0.99	0.84–1.16	158	0.98	0.83–1.15	46	0.91	0.68–1.22	41	1.12	0.82–1.53	36	1.06	0.76–1.48	29	1.05	0.73–1.52	17	1.02	0.63–1.65
*Solid tumors*																									
Bladder	103	4	0.64	0.23–1.77	1	_	_	3	0.83	0.26–2.67	1	_	_	0	_	_	0	_	_	1	_	_	0	_	_
Colon	236	28	1.19	0.80–1.75	18	1.42	0.87–2.32	16	1.17	0.70–1.95	3	0.79	0.25–2.49	6	1.73	0.76–3.91	4	1.24	0.46–3.36	3	1.31	0.42–4.12	4	2.41	0.89–6.53
Glioma	44	**11**	**3.52**	**1.72–7.21**	4	1.81	0.64–5.12	2	_	_	**3**	**4.45**	**1.36–14.55**	1	_	_	2	_	_	2	_	_	0	_	_
Kidney	71	6	0.89	0.38–2.08	3	0.85	0.26–2.72	5	1.41	0.56–3.57	0	_	_	1	_	_	0	_	_	2	_	_	0	_	_
Lung	203	15	0.70	0.41–1.20	10	0.90	0.47–1.71	10	0.84	0.44–1.59	2	_	_	2	_	_	1	_	_	5	2.13	0.87–5.21	2	_	_
Melanoma (cutaneous)	145	12	1.08	0.59–1.97	4	0.63	0.23–1.72	5	0.88	0.36–2.18	2	_	_	3	2.20	0.69–7.02	3	2.40	0.75–7.64	1	_	_	1	_	_
Pancreas	55	7	1.33	0.59–2.97	3	1.03	0.32–3.34	1	_	_	**3**	**3.70**	**1.15–12.0**	1	_	_	1	_	_	0	_	_	0	_	_
Rectum	69	8	1.27	0.60–2.70	6	1.80	0.77–4.21	6	1.79	0.76–4.22	0	_	_	1	_	_	1	_	_	1	_	_	0	_	_
Stomach	26	5	2.61	0.96–7.11	1	_	_	3	2.64	0.76–9.15	1	_	_	1	_	_	0	_	_	0	_	_	0	_	_
Thyroid	54	5	0.66	0.26–1.63	4	0.97	0.36–2.67	1	_	_	0	_	_	1	_	_	1	_	_	0	_	_	0	_	_
*Lymphohematopoietic malignancies*																									
NHL	233	28	1.23	0.82–1.83	17	1.35	0.82–2.22	17	1.35	0.81–2.22	6	1.60	0.71–3.60	1	_	_	3	1.03	0.33–3.24	3	1.49	0.48–4.68	0	_	_
Multiple Myeloma	42	8	2.01	0.91–4.42	**6**	**2.71**	**1.12–6.55**	4	1.75	0.61–5.01	1	_	_	0	_	_	1	_	_	1	_	_	0	_	_
Myeloid Leukemia	34	4	1.26	0.44–3.65	3	1.82	0.55–6.09	3	1.66	0.49–5.56	0	_	_	0	_	_	1	_	_	0	_	_	0	_	_
*Female specific sites*																									
Breast	1,214	99	0.89	0.72–1.09	56	0.93	0.71–1.22	52	0.89	0.67–1.18	17	0.88	0.54–1.42	11	0.88	0.48–1.59	11	0.93	0.51–1.68	5	0.49	0.20–1.18	6	1.06	0.48–2.38
ER + PR+	736	64	0.94	0.73–1.23	36	0.98	0.70–1.37	33	0.93	0.65–1.33	9	0.75	0.39–1.45	8	1.00	0.50–2.02	8	1.05	0.52–2.11	2	_	_	3	0.83	0.27–2.60
ER-PR-	202	15	0.82	0.48–1.40	9	0.90	0.46–1.76	7	0.76	0.36–1.63	4	1.22	0.45–3.30	2	_	_	2	_	_	1	_	_	**3**	**3.55**	**1.12–11.18**
ER + PR-	125	8	0.65	0.32–1.35	5	0.78	0.32–1.92	4	0.61	0.22–1.67	2	_	_	0	_	_	0	_	_	1	_	_	0	_	_
Ovarian	106	9	0.65	0.30–1.61	7	1.05	0.40–2.89	5	0.77	0.20–2.46	2	_	_	2	_	_	2	_	_	1	_	_	1	_	_
Uterine	276	20	0.83	0.50–1.32	10	0.80	0.40–1.50	10	0.82	0.42–1.56	2	_	_	4	1.50	0.60–4.06	3	1.12	0.40–3.51	2	_	_	1	_	_

Significant Findings are listed in boldface

*AHS* Agricultural Health Study, *RR* relative risks, *OC* organochlorines, *ER* estrogen receptor, *PR* progesterone receptor

^±^ *n* ≥ 3 exposed cases

^a^Ever use of any of the seven OC insecticides

^b^Adjusted for age, education, state of residence, pack-years smoked, and alcohol consumption

^c^Inclusive of all reported cancer sites

Although chlordane use (N_exposed_ = 6, RR = 2.71, 95% CI 1.12 to 6.55) was significantly associated with an increased risk for MM, any OC use (N_exposed_ = 8, RR = 2.01, 95% CI 0.91 to 4.42) and DDT use (N_exposed_ = 4, RR = 1.75, 95% CI 0.61 to 5.01) were non-significantly associated with an increased risk of MM (Table 2). There were also several suggestive associations for lymphoematopoietic malignancies. Any OC use was non-significantly associated with an increased risk for NHL overall (N_exposed_ = 28, RR = 1.23, 95% CI 0.82 to 1.83). Similarly, use of chlordane (N_exposed_ = 17, RR = 1.30, 95% CI 0.82 to 2.22), DDT (N_exposed_ = 17, RR = 1.35, 95% CI 0.81 to 2.22), and lindane (N_exposed_ = 6, RR = 1.60, 95% CI 0.71 to 3.60) were also non-significantly associated with increased risks in NHL. We had limited power for NHL subtype analyses. However, all 28 OC exposed cases were B-cell lymphomas. Among women who reported any OC use, there were eight MM cases and six diffuse large B-cell lymphoma (DLBCL); no other B-cell subtype had more than four exposed cases.

We also evaluated hormone-mediated cancers including ovarian, uterine, and breast (see Table 2). No significant associations were found for any OC use or for use of the seven individual OCs and uterine or ovarian cancers. Similarly, we found no association between any OC use or for use of the seven individual OCs and breast cancer. In analyses of breast cancer subtype, there was a statistically significant elevated association between dieldrin use and ER-PR- breast cancer (N_exposed_ = 3, RR = 3.55, 95% CI 1.12 to 11.18).

## Discussion

In this study, we prospectively evaluated associations between the reported personal use of individual OCs and incident cancers in a population of female farm spouses. Although the numbers of exposed cases were small, we observed statistically significant increased risks for use of individual OCs insecticides and several cancers, including any OC use and glioma, lindane use and glioma and pancreatic cancer, chlordane use and MM, and dieldrin use and ER−/PR- breast cancer.

In addition to chlordane, MM was non-significantly associated with any OC use and with DDT specifically. These associations are consistent with previous findings [11–14, 17, 18]. The definition of NHL used in our study is based on the most recent lymphoma classification system, which includes MM as a subtype of NHL [50], whereas most previous studies relied on earlier classifications which considered MM separately. A previous population-based case–control study found non-significant positive associations of ever handling (mixing or applying) aldrin, DDT, or lindane with MM [51]. A pooled analysis of U.S. and Canadian case-controls studies found that DDT use was significantly associated with MM [17]; cumulative exposure to DDT, as measured by lifetime-days of use, was also significantly associated with an increasing risk trend for MM. Although not significant, in the current study, chlordane use was also positively associated with NHL and myeloid leukemia, DDT use was positively associated with NHL and myeloid leukemia, and lindane use was positively associated with NHL. There is evidence that some OCs, including lindane and DDT, cause oxidative stress and immunosuppressive effects, and that these mechanisms possibly play a role in the development of lymphohematopoietic cancers [7, 52–55].

We observed no significant association with Any OC use and breast cancer overall. Although some studies have reported an increased risk of breast cancer among women exposed to OCs during critical developmental windows in early life [41–43], our findings are consistent with most other studies that also did not evaluate timing of exposure [7, 29, 30, 32–39]. Although we did not have information on timing of exposure, we conducted sensitivity analyses using year of birth as a surrogate for the potential for exposure during critical developmental periods. OCs were first registered in 1948, therefore we assumed women born before 1936 would not have any OC exposures prior to menarche. When we restricted analyses to women born after 1936, the RR for breast cancer and any OC use was 1.22 (0.94–1.59) (*n* = 61 exposed cases) compared to 0.84 (0.60–1.18) (*n* = 38 exposed cases) among women who were born prior to 1936. Although there was some evidence to indicate potential differences between the two groups, the test for interaction was not statistically significant (*p* = 0.11. An early study by Wolff et al. found a two- to four-fold increased risk of breast cancer among women with the highest serum DDE levels, with a positive trend with increasing serum DDE [30]. However, a follow-up study with a larger sample size found no evidence for an association of breast cancer risk with serum DDE levels [56]. Additional studies of breast cancer and OC exposures have examined associations with mirex, HCB, and chlordane; most of these studies also reflected null or inconclusive findings [33, 36, 57–59].

We did, however, see an association between dieldrin use and ER−/PR- breast cancer based on only 3 exposed cases. Two previous studies reported positive associations with dieldrin use and breast cancer overall. The first, a Danish case–control study found a significant dose-related increased risk of breast cancer among women and increasing serum concentrations of dieldrin [34]. Additionally, a previous study of AHS farm spouses found evidence for a significant increased risk of breast cancer overall among women who never personally used dieldrin, but whose husbands’ did personally apply the pesticide [35]. This study was unable to assess associations between the wives’ personal use of dieldrin and breast cancer due to the low number of dieldrin exposed breast cancer cases. Our current analysis includes 60 more OC exposed cases and thirteen additional years of follow-up than this previous analysis [35], and was sufficiently powered to examine breast cancer subtypes. Few epidemiologic studies have examined associations between OC exposures and breast cancer subtypes [42, 60–62], and most have not found positive associations with ER-negative breast cancers. In vitro and animal studies have suggested that dieldrin, DDT, endosulfan, HCH, and toxaphene have the potential to elicit tumor promoting effects mediated through the induction of ER, androgen receptor and aromatase activities [46–48, 63, 64]. Given this body of literature and the small number of dieldrin exposed ER-PR- breast cancer cases, our positive finding warrants further investigation. Overall we do not see strong evidence of an association between use of an individual OC and breast cancer, consistent with the existing epidemiologic literature.

Aldrin use was associated with a non-statistically significant elevated risk for uterine cancer based on four exposed cases. Only one case–control study has examined OC exposures and endometrial cancer; no statistically significant associations were observed with several OC derivatives including DDE, oxychlordane, HCH, and HCB [44]. Very few occupational studies have examined the relationship between endometrial cancer and exposure to other OC compounds, including polychlorinated biphenyls (PCBs) [65–67] and the majority of these studies’ findings were null. To our knowledge, this is the first prospective study to examine the relationship between personal use of specific OC insecticides and uterine cancer.

Any OC use and lindane specifically were associated with risk of glioma. While we lacked sufficient power for further subtype analyses, the OC-exposed glioma cases consisted of glioblastomas (*n* = 7), an astrocytoma (*n* = 1), an oligodendroma (*n* = 1), and mixed gliomas (*n* = 2). Previous studies of male farming populations have found some evidence for an increased risk of glioma with associated pesticide use [68–72]. However, studies examining associations between glioma and pesticide exposures among women, in agricultural populations, have provided inconsistent results. In an earlier case–control study of central nervous system cancers among women across twenty-four U.S. states, increased risks were found for women generally exposed to herbicides, insecticides, or fungicides [73]. An analysis of occupational risk factors for glioma found significantly increased risks among women involved in occupations in agricultural services and farming, though this analysis did not examine exposures to specific pesticides [74]**.** However, a case–control analysis of women in Nebraska found no association between individually evaluated OCs (i.e. aldrin, chlordane, DDT, dieldrin, heptachlor and lindane) and brain cancer [68]. Similarly, in a case–control analysis of women in the Midwest, no association was found for gliomas and the personal application of pesticides including OCs [75]. Mechanisms of action for OC-induced gliomas have not been proposed; however, in vitro studies have found that neurotoxic effects induced by the interaction of OCs with ER**-**mediated signaling pathways may play a role [76].

The increased risk of pancreatic cancer associated with lindane use in our study was based on only three exposed cases. Some studies have shown significant increased risks for pancreatic cancer with occupational DDT exposure [26, 27] and significantly higher levels of DDT exposure among pancreatic cancer cases versus controls [27]. However, a previous AHS study found no evidence for an increased risk of pancreatic cancer with the OCs aldrin, DDT, heptachlor or toxaphene [77]. The aforementioned study did not evaluate risk estimates among the spouses only, but examined combined risk estimates among the applicators and their spouses. Furthermore, a lack of exposed cases prohibited the insecticide-specific evaluation of chlordane, dieldrin and lindane. To our knowledge, no other studies have evaluated OC use and pancreatic cancers among women.

Strengths of our study include the prospective longitudinal design with little loss-to-follow-up, questionnaire information on the use of specific OCs, and regular assessment of cancer incidence and mortality via linkage with state registries. The AHS also has detailed information on many possible confounders. Most previous studies of OC exposures and cancer, except for studies of DDT and breast cancer [7, 29–32, 36, 38, 78] have primarily focused on occupationally-exposed men [79]. Our study examined the personal use of DDT, and other specific OCs, in a population of farm women. Few studies have evaluated personal use of specific OCs. While breast cancer has been the most widely studied cancer with respect to OCs, in particular DDT, no studies thus far have prospectively studied OCs and other hormone-mediated cancers.

Limitations of this analysis include the small number of cases exposed to specific OCs and lack of information on duration, time period, and intensity of OC use. While we had a low response rate of the female and family health questionnaire, our reported results and final models were based solely on information collected from the spousal enrollment questionnaire. Questionnaire information was collected at study enrollment (1993–1997), thus changes in individual characteristics (i.e. menopausal status, smoking) since enrollment were not captured in this analysis. In addition, most OCs examined in this analysis have been banned for use in the United States since the 1970s. Because OCs have long half-lives, and are known to persist in the environment and human body for long periods of time [1–3], exposure to OCs through environmental exposure pathways may also contribute to lifetime cumulative exposure. This could be particularly important in farm situations where OCs may have been used in the past.

## Conclusions

We observed significant increased risks for some cancers associated with individual OC insecticides. Despite the large size of the cohort, the numbers of exposed women and cancer cases were small for most cancer sites of interest. While some of our findings are consistent with previous findings, results need replication with longer follow-up time in other studies. Due to the environmental persistence of OCs, future research should attempt to expand on these findings by assessing environmental sources of OC exposures in order to fully evaluate the role of OC exposures on cancer risk in women.

## Abbreviations

AHS: Agricultural health study
CI: Confidence interval
DDT: Dichlorodiphenyltrichloroethane
DLBCL: Diffuse large B-cell lymphoma
HCB: Hexachlorobenzene
HCH: Hexachlorocyclohexane
IARC: International Agency for Research on Cancer
MM: Multiple myeloma
NHL: Non-Hodgkin lymphoma
OCs: Organochlorines
PCBs: Polychlorinated biphenyls
PCP: Pentachlorophenol
RR: Relative risks
SEER: Surveillance, epidemiology, and end results
TCP: 2,4,6-trichlorophenol
DDE: Dichlorodiphenyldichloroethylene
